# Validation of an image analysis algorithm to quantify leukocyte populations using whole slide image analysis

**DOI:** 10.1186/2051-1426-1-S1-P60

**Published:** 2013-11-07

**Authors:** Anthony J Milici, David Young, Steven J Potts, Holger Lange, Nicholas D Landis, Erik R Hagendorn, Sherri A Saturley, Lisa Hall, Joseph S Krueger

**Affiliations:** 1 Flagship Biosciences, Boulder, CO, USA

## 

In recent years, the tumor microenvironment (TME) has been identified as an important factor influencing the growth and metastasis of the tumor. In the TME, different classes of inflammatory cells have been found to exert either a pro- or anti-tumor effect. This has resulted in a growing need to utilize immunohistochemistry to label these leukocyte populations, thereby allowing for the cells to be quantified. In many instances these studies have been performed utilizing 2-3 independent readers to manually quantify the cells requiring significant time both for the actual counting as well as the training needed to minimize the variation from reader to reader. In addition, manual counting is usually done on selective high-powered fields rather than the entire specimen, resulting in variations in counts when different fields are chosen. A key method to increase the throughput and to decrease the variability is to utilize whole slide imaging and computerized image analysis to provide leukocyte counts. An image analysis algorithm which can automatically differentiate tumor from stroma would allow rapid quantification of endpoints in each compartment, such as: tumor burden; number of inflammatory cells/area; or percent of inflammatory cells/total cells in each tissue compartment. In this poster, the validation and utilization of an algorithm to quantify immunolabeled leukocytes in both tumor sections and tissue microarrays is described. Utilizing whole slide imaging approaches, an image analysis algorithm (CellMap™) that allows the quantitation of leukocyte populations (e.g., CD3+, CD8+, FoxP3+) automatically across whole tissue sections has been developed. This approach has been used to evaluate samples of colorectal cancer and non-small cell lung carcinoma. Using this algorithm, leukocyte populations were quantified in sections that have been either singly or dually labeled for inflammatory markers. Accuracy of the algorithm was demonstrated by comparing data from manual counts to algorithm derived counts using high-powered fields. The results of the high-powered field analysis were compared to an analysis across the whole tissue section, demonstrating the effect of variability when user defined fields are chosen. These data support using CellMap™ in the prospective or retrospective assessment of leukocyte subpopulations in clinical samples. This approach will diminish variability in counting, expand the types of endpoints determined, and improve the statistical value of these determinations, thereby facilitating robust TME measurements with clinical value.

